# Lung Involvement in Pulmonary Vasculitis: A Radiological Review

**DOI:** 10.3390/diagnostics14131416

**Published:** 2024-07-02

**Authors:** Luca Gozzi, Diletta Cozzi, Giulia Zantonelli, Caterina Giannessi, Simona Giovannelli, Olga Smorchkova, Giulia Grazzini, Elena Bertelli, Alessandra Bindi, Chiara Moroni, Edoardo Cavigli, Vittorio Miele

**Affiliations:** 1 Department of Experimental and Clinical Biomedical Sciences, Careggi University Hospital, University of Florence, 50135 Florence, Italy; luca.gozzi@unifi.it (L.G.); caterina.giannessi@unifi.it (C.G.); simona.giovannelli@unifi.it (S.G.); olga.smorchkova84@gmail.com (O.S.); 2 Department of Emergency Radiology, Careggi University Hospital, 50134 Florence, Italy; grazzini.giulia@gmail.com (G.G.); elena.bertelli3@gmail.com (E.B.); bindi.alessandra@gmail.com (A.B.); chiaramoroni73@gmail.com (C.M.); edoardocavigli@yahoo.it (E.C.); vmiele@sirm.org (V.M.); 3 Department of Biomedical Sciences for Health, University of Milan, 20133 Milan, Italy; giulia.zanto@gmail.com

**Keywords:** pulmonary vasculitis, small vessel vasculitis, large vessel vasculitis, computed tomography

## Abstract

Pulmonary vasculitis identifies a heterogeneous group of diseases characterized by inflammation, damage and necrosis of the wall of pulmonary vessels. The most common approach to classify vasculitis is according to etiology, therefore dividing them into primary and secondary, with a further sub-classification of primary vasculitis based on the size of the affected vessels (large, medium, and small). Pulmonary involvement is frequently observed in patients with systemic vasculitis and radiological presentation is not pathognomonic, but may vary between diseases. The main findings using high-resolution computed tomography (HRCT) include small vessel wall thickening, nodular lesions, cavitary lesions, reticular opacities, ground-glass opacities (GGO), consolidations, interlobular septal thickening, tracheobronchial stenosis, and aneurysmal dilatation of pulmonary arteries, with or without pleural effusion. Radiological diagnosis alone is difficult since signs and symptoms of lung vessel involvement are often non-specific and might overlap with other conditions such as infections, connective tissue diseases and neoplasms. Therefore, the aim of this review is to describe the most common radiological features of lung involvement in pulmonary vasculitis so that, alongside detailed clinical history and laboratory tests, a prompt diagnosis can be performed.

## 1. Introduction

Vasculitis identifies a heterogeneous group of diseases characterized by blood vessel inflammation, damage, and necrosis of the vascular wall. Pulmonary vasculitis refers to a group of vasculitis affecting the lung or the pulmonary vessels. Following the International Chapel Hill Consensus Conference classification system, vasculitis is divided according to the size of the affected vessels into small vessel vasculitis (the most common pulmonary vasculitis), medium vessel vasculitis, and large vessel vasculitis [1]. Although the Chapel Hill classification has been widely adopted for the description of pulmonary vasculitis, it has been modified by some authors in 2012 to include Behçet’s disease, primary immune complex-mediated vasculitis, and secondary vasculitis (Table 1). Typically, vasculitis presents as difficult-to-diagnose scenarios as they are characterized by nonspecific symptoms. Despite lung involvement being one of the most common aspects in primary vasculitis and in idiopathic vasculitis associated with small vessel antineutrophil cytoplasmic antibodies (ANCA), it is often subtle in term of signs and symptoms, commonly presenting with hemoptysis, cough, dyspnea and asthma [2]. In addition, as it happens in cases of alveolar hemorrhage, an increased carbon monoxide diffusing capacity (DLCO) can be observed, mainly due to the presence of hemoglobin in the alveoli and iron deficiency anemia. Common manifestations of pulmonary vasculitis include alveolar capillaritis with pulmonary hemorrhage, vascular occlusion or thrombosis with lung infarction or ischemia, lung inflammation in association with vascular lesions, and involvement of the large arteries with thrombosis, stenosis, or aneurysm formation. Radiologically, chest X-ray (CXR) is often unspecific, with low sensitivity and specificity, usually leading to underestimation of lung involvement. Therefore, in order to assess the type of lung involvement, the extension and the distribution of the disease, high-resolution computed tomography (HRCT) is the gold standard in patients with respiratory symptoms. However, pulmonary vasculitis radiological patterns are often heterogeneous and non-specific, typically presenting as: small vessel wall thickening, nodular lesions, cavitary lesions, reticular opacities, ground-glass opacities (GGO) with or without a “crazy paving” appearance, consolidations, interlobular septal thickening, tracheobronchial stenosis, and aneurysmal dilatation of pulmonary arteries [3,4] (Table 2 and Table 3). In medium-large vessels vasculitis, contrast enhanced CT (CECT) or magnetic resonance (MRI) might be valid options helping in measuring and detecting vessels narrowing/dilatation. MRI in particular might be useful in younger people, due to the lack of radiation exposure, with the major limits of a low signal-to-noise ratio (SNR), artifacts and lower spatial resolution compared to CECT. Thus, a detailed clinical history, in addition to laboratory findings and a biopsy of the involved tissue, are usually needed to perform a correct diagnosis of vasculitis [5]. For these reasons, the main purpose of this review is to describe the most frequent HRCT pulmonary manifestations of vasculitis, focusing primarily on clinical and radiological features.

## 2. Primary Vasculitis

### 2.1. Small Vessel Vasculitis

Small vessel vasculitis primarily affects small to microscopic arteries, capillaries, and glomeruli. This condition includes various forms of vasculitis, particularly involving the small pulmonary vessels, such as:

### 2.2. ANCA-Associated Vasculitis (AAV)

AAV is characterized by the presence of autoantibodies targeting neutrophil proteins, specifically leukocyte proteinase 3 (PR3-ANCA) or myeloperoxidase (MPO-ANCA). Cytoplasmic ANCA (c-ANCA), determined by anti-PR3 antibodies, is commonly found in granulomatosis with polyangiitis (GPA) in about 90% of cases. Conversely, perinuclear ANCA (p-ANCA) with anti-MPO specificity typically orientates towards microscopic polyangiitis (MPA) or eosinophilic granulomatosis with polyangiitis (EGPA). However, there are instances of ANCA-negative AAV, occasionally seen in eosinophilic granulomatosis with polyangiitis and, to a lesser extent, in granulomatosis with polyangiitis [6]. Pulmonary involvement is a significant risk factor for mortality in patients with AAV. Severe pulmonary manifestations, such as diffuse alveolar hemorrhage and interstitial lung disease, are associated with poor prognosis. High-resolution computed tomography (HRCT) often reveals interstitial lung changes in AAV patients, particularly in those with microscopic polyangiitis and MPO-ANCA positivity [7,8].

### 2.3. Granulomatosis with Polyangiitis (GPA)

**Figure 1:**
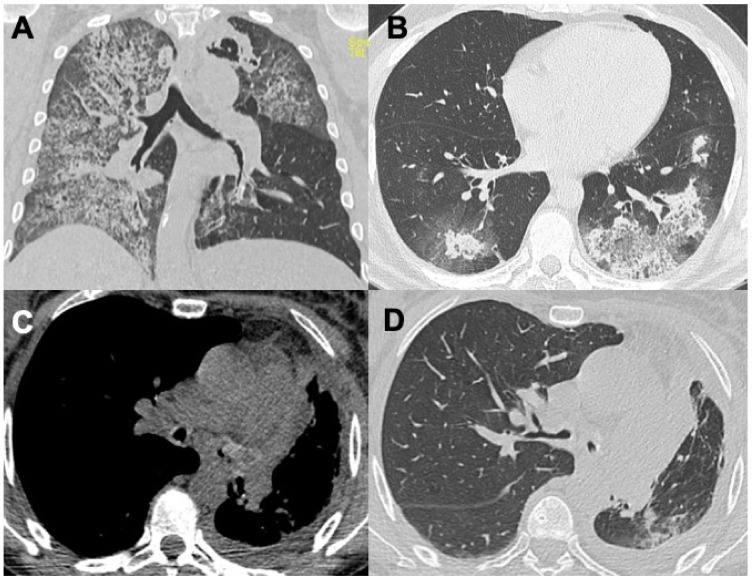
Figures in (**A**,**B**) show two cases of alveolar hemorrhage in GPA, figures in (**C**,**D**) are a case of tracheal and bronchial stenosis due to granulomatous infiltrates.

Granulomatosis with polyangiitis (GPA), previously known as Wegener granulomatosis, is a systemic necrotizing non-caseating granulomatous vasculitis that affects the upper and lower respiratory tracts, often associated with glomerulonephritis (80%) and involvement of the nervous system (polyneuritis), eyes, skin, muscles and joints. GPA is the most common ANCA-related vasculitis, with an estimated incidence of 10–20 cases per million people, an onset typically around 50 years and a slight prevalence in males. Respiratory involvement is common in GPA and patients can be either asymptomatic or symptomatic, presenting with a cough, hemoptysis, chest pain, dyspnea, and tracheal obstruction (Figure 1) [9]. Tracheobronchial involvement is characteristic in GPA, typically in the form of concentric parietal thickening (~70%) often leading to tracheal and bronchial stenosis, mass lesions, tracheobronchomalacia and tracheoesophageal fistulas [10,11,12]. HRCT findings consist of multiple, bilateral pulmonary nodules (~90%) representing necrotizing lesions, usually less than 10 in number, tending to confluence each other and ranging in dimension between a few millimeters and 10 cm; characteristically, these nodules, in particular the larger ones, are prone to cavitate. Rarely, a “tree-in-bud” appearance may be encountered (<10% of cases). Additional findings are represented by septal lines, GGO and consolidations (~50%), representing areas of alveolar hemorrhage or pneumonia and bronchiectasis. Despite the distribution being either peribronchovascular or peripheral, there is not a size or lung zone predilection. Non-cavitated nodules, consolidations and GGO are usually characteristic of mild lung disease, whereas nodule cavitation and associated alveolar hemorrhage are more typical of a late stage disease, representing a potentially life-threatening condition [4,11,13,14,15]. Pulmonary nodules generally respond to immunosuppressive therapies (up to 50% of nodules disappear after treatment), although their evolution often fluctuates over time with the disappearance of some nodules and the appearance of new ones [8]. Pleural effusion is reported in 15% of GPA cases, typically as a consequence of cardiac or renal involvement. Other pleural manifestations, though uncommon, include pleuritis, pleural nodules and pneumothorax [9]. Expiratory HRCT imaging can be useful for detecting areas of air trapping, which might be the only pathological finding in some patients. Pulmonary fibrotic changes occur infrequently, affecting less then 2% of patients [16]. A rare manifestation of GPA, represented by proximal pulmonary artery stenosis, has been reported by Jain et al. [17].

The main radiological differential diagnoses of GPA are hematogenous metastases, septic emboli and lung abscesses. Differential diagnosis is often challenging because the radiological findings are very similar to each other and often indistinguishable (multiple bilateral perivascular nodules ± GGO and cavitations). Therefore, a proper clinical assessment is necessary to make an accurate diagnosis (Table 4).

### 2.4. Microscopic Polyangiitis (MPA) 

**Figure 2:**
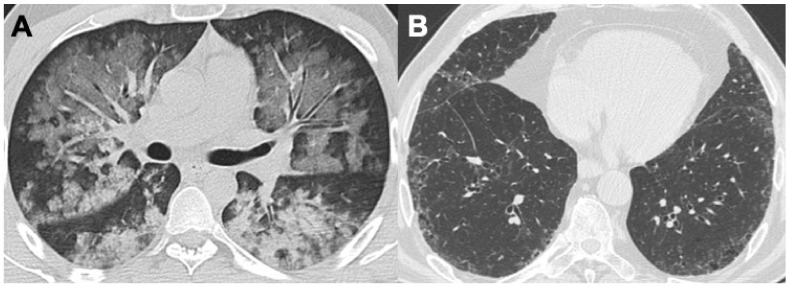
Two cases of MPA. The first (**A**) shows bilateral alveolar hemorrhage with diffuse ground-glass opacities; the second (**B**) presents mild subpleural reticulation with bronchiolectasis in ILD with a UIP-like pattern.

**Figure 3:**
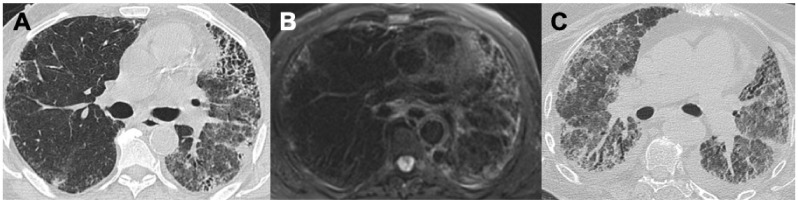
Two cases of patients with ANCA+ vasculitis and pulmonary fibrosis: in particular, the patient in (**A**,**B**) shows a diffuse area of ground glass opacity in the left lung, confirmed with lung magnetic resonance (MR, T2 TSE blade). The patient in (**C**) has an important alveolar hemorrhage and a fibrosis exacerbation, also with pleural effusion.

Microscopic polyangiitis (MPA) is a necrotizing vasculitis that affects arterioles, venules, and alveolar capillaries, characterized by a paucity or absence of immune complexes. Unlike GPA, in MPA the absence of granulomatous inflammation is peculiar. The incidence of MPA is approximately 1.5 per 100,000 people, typically affecting middle-aged individuals, with a slight predilection for males. Glomerulonephritis is the most common manifestation (~90%), while pulmonary diffuse capillaritis is observed in about 30% of cases. Hemoptysis can be the first symptom of alveolar hemorrhage in patients with MPA and might be one of the first reasons for patients to seek further evaluation [18,19]. With HRCT, MPA findings are often unspecific and in keeping with diffuse alveolar hemorrhage (DAH). Therefore, a patchy or diffuse GGO and a “crazy-paving” appearance, mainly with perivascular distribution, are the most frequents patterns occurring in up to 75% of patients. Consolidations, as a consequence of complete alveolar filling, might be an evolution of GGO. Additionally, reticulations and bronchiectasis are observed in 40% of cases [7,20,21]. Moreover, MPA may be associated with interstitial lung disease (ILD). In these cases, lung changes are usually symmetrical and often involve the periphery and lower lung lobes, defining a usual interstitial pneumonia (UIP) pattern (50–71% of cases) (Figure 2 and Figure 3); less commonly, non-specific interstitial pneumonia (NSIP) patterns and desquamative interstitial pneumonia (DIP) patterns are observed, respectively, in 7–31% and 14% of cases [8,22,23]. Pleural effusion may be noted, although pleuritis is rarely described [24]. 

### 2.5. Eosinophilic Granulomatosis with Polyangiitis (EGPA) 

**Figure 4:**
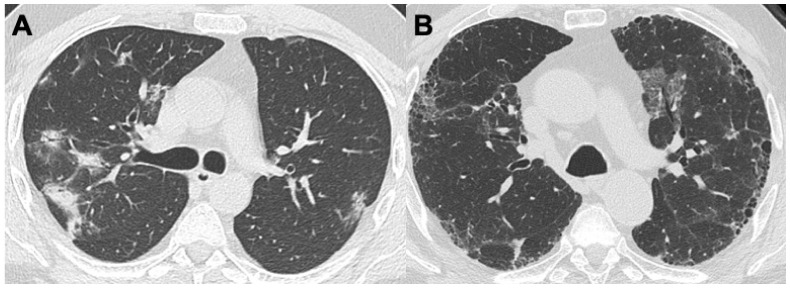
Two cases of pulmonary involvement in EGPA with bilateral alveolar hemorrhage (**A**) and a focal area of bleeding in the left upper lobe in a patient with lung fibrosis (**B**).

Eosinophilic granulomatosis with polyangiitis (EGPA), previously known as Churg-Strauss polyangiitis, is an allergic granulomatous vasculitis typically characterized by the clinic triad of: asthma, peripheral eosinophilia and necrotizing vasculitis. According to the American College of Rheumatology, the diagnosis can be made if at least four of the following six findings are present: asthma, blood eosinophilia >10%, poly- or mononeuropathy, migratory lung opacities, paranasal sinus disease and the histological presence of extravascular eosinophilia. The progression of the disease might be outlined in three phases: a first prodromic phase, characterized by asthma and/or allergic rhinitis; subsequently, a second phase may develop, characterized by eosinophilia and lung involvement. Lastly, the disease may progress to a third phase, in which the heart is affected in the form of coronary arteritis and myocarditis, being the major determinant in mortality. The annual incidence of MPA is between 0.9–2.4 per million, without a gender predilection and a mean age of onset around 35–40 years [25]. Pulmonary involvement in microscopic polyangiitis (MPA) is clinically characterized by severe asthmatic episodes and the presence of pulmonary eosinophilic infiltrates. HRCT findings typically reflect these infiltrates: consolidations and GGO are the most common imaging findings, occurring in approximately 90% of cases. These are typically transient and have a peripheral and/or peribronchovascular distribution. Other HRCT findings include bronchial wall thickening, centrilobular nodules with a “tree-in-bud” appearance, and thickening of the interstitium due to edema or eosinophilic infiltrates (Figure 4). Additionally, pleural effusion (of a cardiogenic nature) might be present in up to 40% of cases [4,26]. Buschman et al. described enlargement of the peripheral pulmonary arteries in association with a stellate-shape and an irregular pattern of some pulmonary arteries [27]. In patients with a history of asthma and lung consolidations, due to the wide variability of radiological presentations, EGPA has a differential diagnosis with other conditions such as organizing pneumonia (OP) and chronic eosinophilic pneumonia (CEP), allergic bronchopulmonary aspergillosis, and an NSIP pattern; therefore, a detailed clinical history of the patients must be collected and EGPA should be considered in the presence of late-onset asthma, systemic manifestations (such as cutaneous rash and peripheral neuropathy) and positivity for ANCA-ab.

### 2.6. Isolated Pauci-Immune Pulmonary Capillaritis (IPIPC) and Immune-Complex Small Vessel Vasculitis

IPIPC is a rare small vessel vasculitis confined to the lungs that causes diffuse alveolar hemorrhage (DAH). It is classically considered a subgroup of MPA, occurring mostly without ANCA positivity. Diffuse alveolar infiltrates are the main HRCT feature; however, bilateral, random, and variable-sized cavitated nodular lesions occasionally might be found [28,29]. 

Immune complex small vessel vasculitis refers to vasculitis with immunoglobulin and/or complement deposits on the vessel wall predominantly affecting the small vessels. Medium-sized arterial involvement is rarer in immune complex vasculitis than in AAV.

Immunoglobulin A vasculitis (IgA vasculitis, also known as Henoch–Schönlein purpura) is a small vessel vasculitis characterized by IgA1 immune complexes tissue deposition, resulting in palpable purpura, arthralgias, enteritis and glomerulonephritis. The disease appears to peak in incidence in spring and is commonly observed in children, with a male-to-female ratio of 1.5:1 [30]. Lung involvement is a rare complication of IgA vasculitis and diffuse alveolar hemorrhage is the main finding from HRCT [31,32]. Rarely, pulmonary manifestations such as interstitial pneumonia, bilateral nodules and pleural effusion may be seen [33,34].

**Figure 5:**
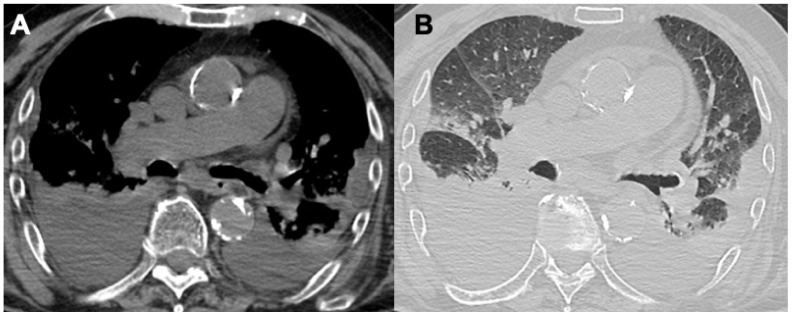
A case of Goodpasture syndrome in a patient with alveolar hemorrhage and cardiac failure with bilateral pleural effusion. In (**A**) mediastinal window shows the presence of pleural-pericardial effusion and (**B**) is characterized by diffuse signs of pulmonary congestion with consolidations and ground glass area.

Goodpasture’s syndrome (GS) is a small-vessel vasculitis characterized by the deposition of antiglomerular basal membrane autoantibodies (anti-GBM) in the glomerular and alveolar basement membranes, leading to glomerulonephritis and alveolar hemorrhage (classic triad: glomerulonephritis, alveolar hemorrhage, and anti-GBM positivity) [35]. Patients with Goodpasture syndrome present with a mean age of onset ranging between 20 and 30 years, with a predominance twice as high in males than females. Typically, symptoms are related to parenchymal involvement in the form of diffuse alveolar hemorrhage (DAH), that may either precede or coincide with glomerulonephritis. According to HRCT findings, the disease may be divided into an acute form, characterized by bilaterally patchy, diffuse or centrilobular GGO and/or consolidations (lung manifestations of DAH), usually with a central distribution and peripheral sparing (Figure 5); and a subsequent subacute phase may develop, presenting superimposition of inter- and intra-lobular septal thickening to GGO ± mediastinal and hilar lymphadenopathy. GGO often resolves in 2–3 weeks with adequate treatment, however the disease may recur or chronicize, developing fibrosis with reticulation, traction bronchiolectasis ± mild honeycombing [36,37]. The most difficult differential diagnoses are with MPA (sharing kidney involvement and DAH), systemic lupus erythematosus (immune complex-mediated small vessel vasculitis, renal involvement in 60–90% of cases and pleural effusion in 50% of cases, with the latter being rare in Goodpasture syndrome), noncardiogenic pulmonary edema (acute onset with diffuse GGO and consolidations) and idiopathic pulmonary hemosiderosis (diffuse and recurrent pulmonary hemorrhage with hemoptysis, but without renal involvement).

Hypocomplementemic urticarial vasculitis (HUV) is a rare systemic vasculitis of unknown etiology that affects small vessels, clinically characterized by urticarial lesions, various systemic manifestations and hypocomplementemia due to the presence of anti-C1q antibodies that at a pulmonary level may cross-react with pulmonary surfactant apoproteins, causing pulmonary symptoms [38]. It has been reported to accompany certain drug assumption, infections (including COVID-19), autoimmune diseases, and malignancy. Typically, pulmonary manifestations occur in 20–50% of patients, clinically presenting with a cough, dyspnea, hemoptysis, chronic obstructive pulmonary disease (COPD), asthma, tracheal stenosis, and pleuritis [38,39]. Additionally, small nodules that are randomly distributed, supraclavicular and axillary adenopathy and splenomegaly may be seen [40].

Cryoglobulinemic vasculitis (CryoVas) is a rare condition characterized by inflammation of the small- and medium-caliber blood vessels, accompanied by the presence of circulating cryoprecipitate immune complexes in the serum. Hepatitis C virus (HCV) infection is identified as the causative factor in 80–90% of cases, often in conjunction with other genetic and environmental factors; however, CryoVas only develops in around 5% of individuals with chronic HCV infection. The prevalence of CryoVas is higher in Southern Europe compared to North America, estimated to be around 1 per 100,000 individuals, with a female-to-male ratio of 2–3:1. With HRCT, bilateral lung infiltrates and signs of alveolar hemorrhage are the most common findings, but rarely some cases of OP have been reported [41,42,43]. 

## 3. Medium Vessel Vasculitis

Medium vessel vasculitis includes vasculitis predominantly affecting “medium-sized” arteries such as those arising from the celiac trunk, the coronary arteries, or the bronchial arteries.

Polyarteritis nodosa (PAN) is a systemic necrotizing vasculitis that primarily affects small to medium-caliber muscular arteries, including renal and visceral arteries. Pulmonary arteries are typically spared in PAN, but bronchial vessels may be involved [44,45]. PAN typically peaks between the ages of 40 and 50 and is more common in males. While many cases of PAN are idiopathic, hepatitis B virus (HBV) infection in particular, hepatitis C virus (HCV) infection, and hairy cell leukemia are recognized as contributing factors in some cases. 

Vascular lesions in PAN are segmental and transmural, predominantly occurring at the bifurcations of medium-sized muscular arteries, leading to lumen narrowing, thrombosis and aneurysmal dilatation of the affected vessel [46]. From HRCT, bilateral lobular and perilobular (arcade-like sign) GGOs can be observed at the branching vessels, both in central and peripheral lung regions. Despite being a rare condition, DAH may also occur [47].

## 4. Large Vessel Vasculitis

Large-vessel vasculitis includes vasculitis that predominantly involve the aorta and its major branches, but may also include other vessels such as pulmonary arteries [48]. The major forms of large vessel vasculitis that involve the pulmonary artery are Takayasu arteritis (TAK) and giant cell arteritis (GCA). 

**Figure 6:**
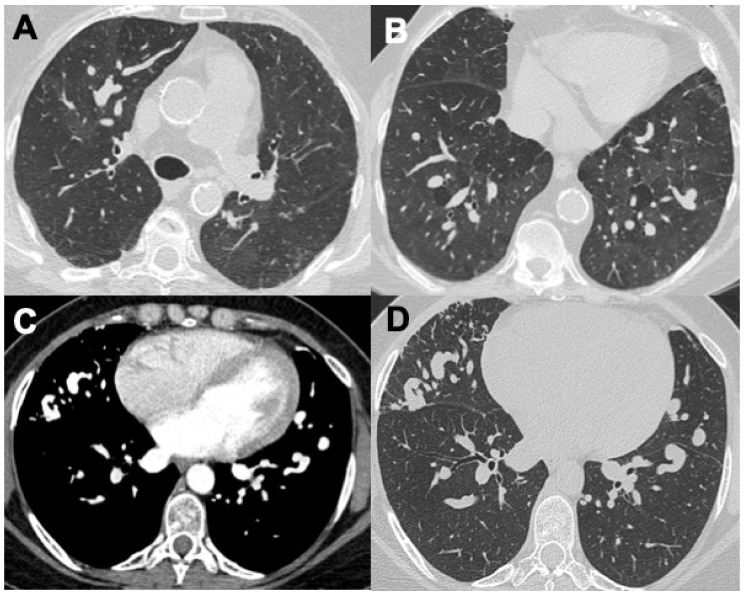
A case of Takayasu arteritis, involving pulmonary arteries: In (**A**,**B**), mosaic attenuation as the result of multiple hypoperfused areas is shown, with a typical ground-glass appearance. In (**C**,**D**), stenosis and post-stenotic dilatation of the arterial pulmonary vessels is shown.

TAK is an inflammatory disease of the medium- and large-caliber arteries, particularly affecting the aortic arch and its branches, causing stenosis, occlusion, or aneurysmal degeneration. It is a rare disease, with an annual incidence of 1–2 cases per million. It predominantly affects young female, with a F:M ratio of 9:1 and a typical age of onset between 15 and 30 years, with a stronger predominance in Asian population [49]. The disease is characterized by granulomatous inflammation of the vessel wall, with intimal proliferation and fibrosis of the tunica media and adventitia. Clinically, TAK can be divided into three phases, reflecting radiological findings. The first phase, or pre-pulseless phase, is clinically characterized by fever, fatigue, and weight loss, while radiologically it is determined by initial vessel wall thickening with or without contrast enhancement; the second phase, or vascular inflammatory phase, is characterized by vessel regurgitation and radiologically by vessel wall late contrast enhancement; lastly, the third phase or occlusive pulseless phase, clinically presents with diminished/absent pulses, hypertension and neurological symptoms, with radiological findings such as vessels stenosis, aneurysms, wall calcification and occlusions. In this last stage, pulmonary hypertension may occur (Figure 6) [50]. Pulmonary artery involvement, typically segmental and subsegmental branches, is observed in 50–80% of cases, causing dyspnea and atypical chest pain [51]. Alterations of the right branch of the pulmonary artery are more commonly observed than those affecting left branches; additionally, the upper and lower lobe branches are more frequently involved compared to middle lobar branches [52]. A patchy decrease in the attenuation of peripheral lung parenchyma is often the consequence of stenosis or obstruction of the segmental or smaller pulmonary arteries, leading to parenchymal hypoperfusion. Positron emission tomography (PET) is an increasingly used test in the diagnosis of TAK as it allows the identification of “hot” vascular segments, suggestive of active vasculitis [53].

Giant cell arteritis, also known as Horton’s (or temporal) arteritis, is an inflammatory process affecting medium- and large-caliber arteries. GCA is the most common primary systemic vasculitis, characteristically affecting one or more branches of the extracranial carotid artery, particularly the temporal artery; however, systemic vasculitis of the large- and medium-caliber arteries are often present. GCA is closely associated with polymyalgia rheumatica, occurs in individuals older than 50 years (typical onset between 70 and 80 years), and more frequently in women. Characteristic symptoms consist of tender and swollen temporal arteries, temporal headache, claudication of the jaw, and loss of vision [54,55]. CT findings of GCA may resemble TAK with arterial wall thickening, stenosis, thrombosis, and aneurysms, with a greater predisposition in dissection depending on whether the aortic arch is involved. Pulmonary involvement in GCA is rare; the unusual involvement of the bronchial arteries was shown in the necropsy of a patient with disseminated GCA [56]. Interstitial lung disease (ILD) may be observed in the form of a UIP-pattern or indeterminate pattern, whereas pleural effusion is rare [57,58]. 

## 5. Variable Vessel Vasculitis 

### Behçet Disease (BD) and Hughes–Stovin Syndrome (HSS)

**Figure 7:**
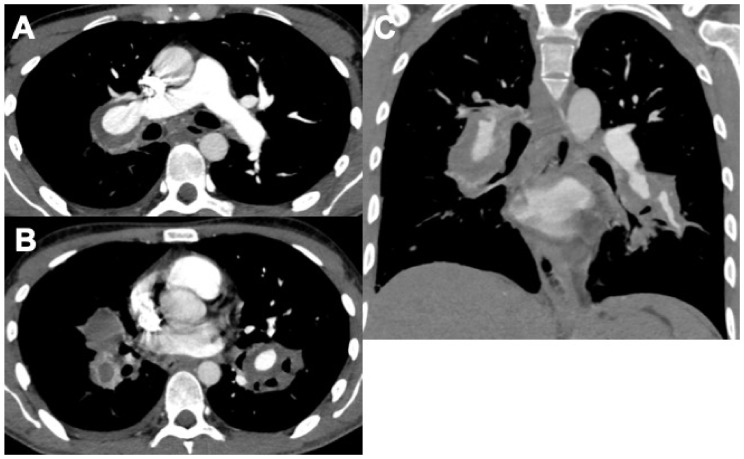
A case of pulmonary arterial involvement in Beçhet’s syndrome, showing large bilateral pulmonary artery aneurysms with eccentric thrombosis, in a young male with massive hemoptysis who arrived at the emergency department. The figures are a CT pulmonary angiography (CTPA) study: (**A**,**B**) in the axial plane and (**C**) in the coronal one.

Behçet’s syndrome is a systemic disease characterized by recurrent ulcers in the oral cavity and genitals mucosa, associated with ocular involvement. It predominantly affects young individuals (20–30 years) from the Mediterranean, Middle, and Far East regions, with a male to female ratio of 3:1 (data subject to regional variations) [59]. The vascular disease develops in up to 40% of cases and large vessel involvement occurs in approximately one-third of patients with BD, affecting both the arterial and venous systems. Venous disease, resulting in venous thrombosis, is the most common vascular manifestation and is often an early feature of BD. Arterial involvement is rarer, presenting with aortitis or peripheral arterial aneurysms or arterial thrombosis. In 5% of cases, pulmonary artery vasculitis is present, clinically manifesting with dyspnea, a cough, chest pain, hemoptysis, and pulmonary infiltrates. Pulmonary artery aneurism is a characteristic feature of BD, and its rupture can lead to the formation of a pulmonary bronchial vascular fistula, which can cause intrapulmonary hemorrhage and even death (Figure 7) [60,61]. Parenchymal involvement is uncommon and may manifest as consolidations or nodules, which may evolve into excavation, peripheral/subpleural wedge-shaped GGO or consolidations (attributable to parenchymal infarction), pleuro-parenchymal fibrotic changes including loss of lung volume, pleural, peri-bronchial, bronchial and interlobular septal thickening [62,63]. Moreover, pleural and pericardial nodules and effusion, as well as mediastinal lymphadenopathy or mediastinitis may be seen [64,65].

Hughes–Stovin syndrome (HSS) is a potentially fatal disease, considered a clinical cardiovascular variant of Beçhet’s disease (BD). It is characterized by the presence of multiple pulmonary and/or bronchial artery aneurysms and deep venous thrombosis, predominantly affecting young men. The disease typically progresses through three sequential phases: an initial systemic thrombosis/thrombophlebitis, followed by the formation and enlargement of aneurysms, and potentially culminating in aneurysm rupture [66,67]. In HSS-related pulmonary vasculitis, underlying vasculitis contributes to intra-aneurysmal in situ thrombosis and vessel wall thickening [68]. Potential radiological findings include systemic thrombi in the vena cava, cerebral sinuses or limb veins, pulmonary arterial occlusions due to emboli or thrombi, multiple aneurysmal dilatations, especially of the bronchial and pulmonary arteries, and pulmonary infarctions [67,68,69]. 

## 6. Secondary Vasculitis

Secondary vasculitis may be triggered by infections, drugs, malignancies, connective tissue diseases (mostly systemic lupus erythematosus), or environmental exposure [70]. The classic triad for diffuse alveolar damage includes hemoptysis, diffuse alveolar infiltrates, and a drop in hematocrit. Typically, diffuse and bilateral lung infiltrates with GGO and crazy-paving patterns are seen on imaging, although these changes may occasionally be unilateral [14,71]. GGO transforming into consolidations indicates the complete hemorrhagic filling of the airspaces. Following episodes of pulmonary hemorrhage, reticular patterns with traction bronchiectasis and honeycombing may develop [55]. According to the Revised Chapel Hill Consensus Conference, secondary vasculitis is categorized into vasculitis associated with systemic disease and vasculitis associated with probable etiology [1]. 

## 7. Vasculitis Associated with Systemic Disease

Vasculitis may be associated with or caused by a systemic disease (more often collagen diseases). 

Rheumatoid pulmonary vasculitis (RPV) is an extra-articular manifestation of rheumatoid arthritis, characterized by the progressive narrowing of small and medium-sized blood vessels. It commonly affects the skin, fingers, eyes, peripheral nerves, and heart, typically occurring in patients with long-standing, active diseases and positive serological tests for rheumatoid factor [72]. Pulmonary involvement in RPV is classically visible on HRCT imaging as diffuse alveolar damage. Interestingly, the study by Tourin et al. identified the presence of multiple nodules and thick-walled cavities in the lungs of the affected patients [73]. 

Vasculitis occurs in 11–36% of cases in systemic lupus erythematosus (SLE) patients [74]. Typically, the vasculitis affects the small vessels, with cutaneous involvement as a typical rash, although medium-sized vessels may also be affected [75]. An association between antiphospholipid antibody syndrome (APS) and lupus vasculitis is reported, and in such cases, the coexistence of vasculitis and APS is associated with a poor prognosis. Pulmonary vasculitis in SLE is rare and is often linked to renal involvement. Diffuse alveolar damage is the main pulmonary manifestation, presenting as GGO that may evolve into consolidations as the alveoli progressively fill, potentially being a life-threatening condition. Additionally, pulmonary hypertension may occur as a consequence of pulmonary vasculitis [76].

Systemic vasculitis is an unusual complication of sarcoidosis; however, pulmonary vascular involvement of small and medium-sized vessels is frequent in patients with sarcoidosis. Sarcoidosis is a multisystem granulomatous disorder of unknown etiology characterized by the presence of noncaseating granulomas, potentially involving every organ, however, showing a particular tropism for lungs, lymph nodes, skin, and eyes. Histologically, perivascular granulomas are recognized around veins and arteries causing compression and distortion, without vascular necrosis [77]. The involvement of large vessels, particularly the aorta and its main branches, may cause arterial wall stenosis or dilatation, and such involvement is often associated with a poor prognosis. Hilar bilateral adenopathy and pulmonary infiltrates, in the form of small perilymphatic nodules, are the most common findings with HRCT examination. Small vessel involvement is occasionally reported in a variant form of sarcoidosis known as necrotizing sarcoid granulomatosis (NSG) [78]. 

NSG is a rare disease considered to be either a variant of sarcoidosis or a primary pulmonary angiitis. It is characterized by necrotizing granulomas coexisting with small and large pulmonary arteries and veins. The disease is more prevalent in females, with an average onset of 42 years old [79]. From HRCT, lung involvement may simulate a pulmonary neoplasm, as the radiological findings typically include nodules or masses with or without cavitation [80]. Other CT findings include pulmonary infiltrates, and pleural thickening [81]. However, mediastinal adenopathy is less common in NSG compared to pulmonary sarcoidosis [77].

## 8. Vasculitis Associated with Probable Etiology

Vasculitis may also be associated with specific etiologies, as found in COVID-19-associated vasculitis, HCV-associated cryoglobulinemic vasculitis, HBV-associated vasculitis, syphilis-associated aortitis, drug-associated immune complex vasculitis, drug-associated ANCA-associated vasculitis (the most common cause is hydralazine), cancer-associated vasculitis and many others. One of the most interesting associations, especially in light of what has happened in recent years, is the one with SARS-CoV-2 infection.

Severe acute respiratory syndrome coronavirus 2 (SARS-CoV-2) infections may be associated with the development of vessel wall inflammation occurring in arteries, arterioles, capillaries, venules, and veins, associated with extensive luminal vascular thrombosis, pulmonary embolism, and pulmonary hemorrhage [82,83]. The topic is still controversial and subject to many discussions, however several examples of pathological pulmonary changes, later labeled as vasculitis, have been described as related to SARS-CoV-2 pneumonia. The multisystem nature of severe COVID-19 infection, coupled with the presence of various autoantibodies, including anti-neutrophil cytoplasmic antibodies (ANCA), can complicate the diagnosis. Moreover, the overlap of symptoms and laboratory findings between COVID-19 and autoimmune conditions make distinguishing between the two challenging. As stated in the study by Kadkhoda et al., 57% of randomly selected patients hospitalized as positive with COVID-19 have tested positive for ANCAs, of which 72% were C-ANCA and 28% were P-ANCA. Additionally, as reported by Giryes et al., very few cases of ANCA-associated vasculitis with a temporal association with COVID-19 infection have been described up to now, showing some irreversible organ damage, but all of them survived after receiving immunosuppressive therapy [83,84]. Furthermore, a case reported by Morris et al., showed a patient tested c-ANCA positive with COVID-19 pneumonia, with imaging revealing bilateral patchy consolidations, suggestive of DAH, and cavitary lesions, suggestive of multifocal pneumonia [85].

Dilatation and tortuous vessels in the subpleural lung regions, close to areas of ground-glass opacity, may be considered the result of damage caused by pro-inflammatory factors or hyperemia induced by SARS-CoV-2 infection [86,87]. However, despite HRCT findings in the acute phase of COVID-19 showing peripheral, bilateral, predominant basal GGOs and consolidations, interlobular septal thickening, crazy paving patterns, and bronchiectasis, identifying the specific radiological findings of vasculitis could be difficult. Lymphadenopathy, pleural and pericardial effusion may also be observed [14,88,89]. Diffuse alveolar damage and thrombosis in small and mid-sized pulmonary arteries may occur in late-stage disease and has been reported in post-mortem examination. In this context, Fogarty et al. suggested a microvascular pathogenic condition known as “pulmonary intravascular coagulation” (PIC), which leads to pulmonary micro-thrombosis and is responsible for COVID-19-related DAD/ARDS [90,91,92]. Furthermore, some rare cases of COVID-19 vasculitis, resembling GCA, Goodpasture syndrome and IgA vasculitis, have been described in the literature, suggesting a possible relation between these conditions and a common radiological presentation [83]. To summarize, COVID-19-related pulmonary vasculitis is a new and evolving topic; however, the proper pathogenic mechanism of vasculitis involvement, as well as representative radiological findings, have yet to be described.

Hydralazine is an antihypertensive and vasodilator drug that has been associated with two drug-induced systemic syndromes: drug-induced lupus and drug-induced ANCA-associated vasculitis. In the latter, the clinical presentation is often associated with pulmonary and renal disease. With CT, bilateral pulmonary interstitial infiltrates and pleural and/or pericardial effusion may be shown [93].

## 9. Pulmonary Angiitis and Granulomatosis

Some granulomatous pulmonary conditions may be associated with a vascular component of pulmonary angiitis. This group includes at least five entities, such as GPA, EGPA, NSG together with lymphomatoid granulomatosis and bronchocentric granulomatosis.

Lymphomatoid granulomatosis (LYG), also known as angiocentric lymphoma and angiocentric immunoproliferative lesion, is a rare lymphoproliferative disorder caused by the Epstein–Barr virus (EBV), usually presenting in adults in their fourth to sixth decade of life [94]. Lung involvement occurs in 80% of cases and the main CT features include small (<1 cm), bilateral, coalescent pulmonary nodules, together with large necrotic lesions that may cavitate. Nodules are generally located in the lower lung areas, along the bronchovascular structures or interlobular septa [95,96]. In addition, thin-walled cysts and enlarged mediastinal lymph nodes may be seen. Pulmonary artery wall involvement may present either with wall thickening or as intraluminal filling defects [97,98]. Respiratory failure, pulmonary embolism, and massive hemoptysis are potentially life-threatening conditions in advanced LYG.

Bronchocentric granulomatosis (BCG) is a rare chronic condition characterized by granulomatous destruction of the walls of bronchi and bronchioles [99]. In 50% of cases, BCG is associated with asthma and allergic bronchopulmonary aspergillosis (ABPA) [100]. With HRCT, typical findings include nodular mass-forming or lobar consolidations with atelectasis, usually unilateral with a preference for upper lobes [97,101]. Mucoid impaction in the bronchi may be seen in patients with consolidation, suggesting ABPA. Frequently, pulmonary arteritis adjacent to the areas of inflammation is observed [102]. 

## 10. Conclusions

Pulmonary vasculitis forms a heterogeneous group of diseases, occurring as a primary process or secondary to another underlying disease, characterized by blood vessel inflammation, damage, and necrosis of the vascular wall, presenting with various CT patterns and appearances. The diagnostic evaluation of a case of possible vasculitis requires a comprehensive approach, including: a detailed history, comprising drug use, exposure to infectious diseases and symptoms of systemic manifestations; laboratory tests, which are essential for identifying underlying causes or associated conditions; and HRCT of the chest, which is crucial for recognizing characteristic imaging features. Radiological findings in pulmonary vasculitis can often overlap, and there may be an absence of pathognomonic signs for specific diseases. Therefore, radiologists must be familiar with the various features of pulmonary vasculitis, especially in acute presentations, to enable prompt diagnosis and establish an appropriate clinical–therapeutic pathway for the patient. Understanding the diverse CT patterns—ranging from ground-glass opacities and consolidations to nodules and cavitations, along with potential vascular involvement—enables radiologists to contribute effectively to the multidisciplinary management of these complex conditions.

## Figures and Tables

**Table 1:** Classification of pulmonary vasculitis following the Revised International Chapel Hill Consensus Conference. GPA: Granulomatosis with polyangiitis; MPA: microscopic polyangiitis; EGPA: eosinophilic granulomatosis with polyangiitis; HUV: hypocomplementaemic urticarial vasculitis; CryoVas: cryoglobulinemic vasculitis.

Primary Vasculitis			
Small vessel vasculitis	ANCA-associated vasculitis: GPA MPA EGPA	Isolated Pauci-immune pulmonary capillaritis	Immune-complex small vessel vasculitis: IgA vasculitis Goodpasture syndrome HUV CryoVas
Medium vessel vasculitis	Polyarteritis nodosa		
Large vessel vasculitis	Takayasu arteritis	Giant cell arteritis	
Variable vessel vasculitis	Behçet disease	(Hughes–Stovin syndrome)	
Secondary Vasculitis			
Vasculitis associated with systemic disease	Rheumatoid Pulmonary vasculitis	Lupus vasculitis	Sarcoid vasculitis
Vasculitis associated with probable etiology			

**Table 2:** Airway, pulmonary and vascular manifestations in ANCA-associated vasculitis. GPA: Granulomatosis with polyangiitis; MPA: microscopic polyangiitis; EGPA: eosinophilic granulomatosis with polyangiitis; GGO: ground-glass opacities; UIP: usual interstitial pneumonia; NSIP: non-specific interstitial pneumonia; and DIP: desquamative interstitial pneumonia.

	Lower Airway Manifestations	Pulmonary Manifestations	Pleural Manifestations	Vascular Manifestations
**GPA**	Tracheobronchial involvement (15–55%); bronchiectasis	Nodules (up to 50%); consolidations; GGO (25–50%) and a “crazy-paving” appearance; diffuse alveolar hemorrhage (22–30%)	Pleural effusion (15%); pleuritis; pneumothorax	Pulmonary artery stenosis
**MPA**	Bronchiectasis (40%)	GGO (23–94%); reticular changes (UIP, NSIP, and DIP patterns); consolidations; diffuse alveolar hemorrhage (25–60%)	Pleural effusion; pleuritis	
**EGPA**	Bronchial wall thickening; mucoid impaction, bronchiectasis	Tree-in-bud opacities; GGO; consolidations; diffuse alveolar hemorrhage (4%)	Pleural effusion	Dilatation of peripheral pulmonary arteries

**Table 3:** Lung involvement in pulmonary vasculitis. GPA: Granulomatosis with polyangiitis; MPA: microscopic polyangiitis; EGPA: eosinophilic granulomatosis with polyangiitis; HUV: hypocomplementemic urticarial vasculitis; BD: Behçet disease; NSG: necrotizing sarcoid granulomatosis; LYG: lymphomatoid granulomatosis; RPV: rheumatoid pulmonary vasculitis; CryoVas: cryoglobulinemic vasculitis; PAN: polyarteritis nodosa; SLE: systemic lupus erythematosus; and BCG: bronchocentric granulomatosis.

Pulmonary Involvement		
**Solid nodules**	GPA (most common) EGPA HUV BD	RPV NSG LYG
**Cavitated nodules**	GPA (most common) BD	RPV NSG LYG
**Tree-in-bud**	EGPA (common) GPA (rarely)	
**GGO**	EGPA (most common) MPA (common) GPA IgA vasculitis (rare) COVID (common)	GS (acute phase) CryoVas PAN (arcade-like sign) BD SLE
**Consolidations**	EGPA (most common) GPA (most common in children) MPA GS (acute phase)	BD SLE COVID BCG
**Reticulations**	GPA MPA (UIP pattern in particular) COVID	GS (late phase) GCA (rare) PAN
**Crazy-paving**	MPA (most common) GPA EGPA COVID	IgA vasculitis (rare) GS (late phase) PAN
**Bronchial thickening**	GPA EGPA	
**Pleural effusion**	EGPA (common) GPA (uncommon)	BD COVID

**Table 4:** Main differential diagnoses of the most common pulmonary vasculitis.

Most Common Pulmonary Vasculitis	Main Differential Diagnoses
**Granulomatosis with polyangiitis (GPA)**	hematogenous metastases septic emboli lung abscesses
**Eosinophilic granulomatosis with polyangiitis (EGPA)**	organizing pneumonia (OP) chronic eosinophilic pneumonia (CEP) allergic bronchopulmonary aspergillosis ILD with an NSIP pattern
**Microscopic polyangiitis (MPA)**	EGPA GPA GS Interstitial lung disease
**Behçet disease (BD)**	Sarcoidosis HSS TAK Pulmonary hemorrhage

## Abbreviations

AAV: ANCA-associated vasculitis; ABPA: allergic bronchopulmonary aspergillosis; ANCA: antineutrophil cytoplasmic antibodies; APS: antiphospholipid antibody syndrome; BCG: bronchocentric granulomatosis; BD: Behçet disease; COPD: chronic obstructive pulmonary disease; CryoVas: cryoglobulinemic vasculitis; DAH: diffuse alveolar hemorrhage; DIP: desquamative interstitial pneumonia; DLCO: diffusion carbon monoxide capacity; EGPA: eosinophilic granulomatosis with polyangiitis; GCA: giant cell arteritis; GGO: ground-glass opacities; GPA: granulomatosis with polyangiitis; HBV: hepatitis B virus; HCV: hepatitis C virus; HRCT: high-resolution computed tomography; HSS: Hughes–Stovin syndrome; HUV: hypocomplementemic urticarial vasculitis; ILD: interstitial lung disease; IPIPC: isolated pauci-immune pulmonary capillaritis; LYG: lymphomatoid granulomatosis; MPA: microscopic polyangiitis; MPO-ANCA: myeloperoxidase antineutrophil cytoplasmic antibodies; NSG: necrotizing sarcoid granulomatosis; NSIP: non-specific interstitial pneumonia; PAN: polyarteritis nodosa; PET: positron emission tomography; PR3-ANCA: protein leukocyte proteinase 3 antineutrophil cytoplasmic antibodies; SLE: systemic lupus erythematosus; TAK: Takayasu arteritis; UIP: usual interstitial pneumonia.
